# The association between smartphone addiction and thumb/wrist pain: A cross-sectional study

**DOI:** 10.1097/MD.0000000000019124

**Published:** 2020-03-06

**Authors:** Ayman Baabdullah, Diyaa Bokhary, Yousof Kabli, Omar Saggaf, Motaz Daiwali, Amre Hamdi

**Affiliations:** a Faculty of Medicine; b Department of Orthopedic Surgery, Faculty of Medicine, King Abdulaziz University, Jeddah, Kingdom of Saudi Arabia.

**Keywords:** addictive, behavior, evaluation studies as topic, pain, smartphone

## Abstract

Many smartphone users experience pain in the thumb/wrist. This pain can be due to certain types of hand injuries as well as inflammation of the extensor pollicis brevis and the abductor pollicis longus tendon sheaths, known as De Quervain tenosynovitis. The objective of this study was to evaluate the association between smartphone addiction and wrist/thumb pain and to determine the severity of the pain, as well as to calculate the prevalence of De Quervain tenosynovitis among medical students at King Abdulaziz University (KAU) in Jeddah.

A total of 387 medical students were enrolled. The smartphone addiction scale-short version (SAS-SV) was used to divide participants into the smartphone addict group and non-addict group. Both groups completed the self-administered patient-rated wrist and hand evaluation (PRWHE) questionnaire to evaluate wrist/hand pain. The Finkelstein test was administered to those who reported pain in the thumb/wrist.

Two hundred fifty-seven (66.4%) participants were smartphone addicts; 74 (19.1%) had a positive Finkelstein test. There was a significant correlation between smartphone addiction and high PRWHE scores (*P* = .036).

Our study found the prevalence of smartphones addiction among university students to be high (66%), furthermore a correlation between heavy smartphones usage and hand pain was found which indicates that heavy usage of these devices can cause subclinical effects on the human hand.

## Abstract 2

Supplemental Digital Content is available in the text

## 1 Introduction

Smartphone devices have evolved rapidly in both function and propagation in the past 2 decades.^[1]^ Smartphones combine the normal mobile phone features with other personal digital assistance functions,^[2]^ including internet browsing, accessing email, global positioning system (GPS) navigation, desktop synchronization, voice recognition, capturing high-quality photos, touchscreen, motion sensor, large displays, and third party applications known as “apps.”^[2]^

Behavioral addiction is described as a failure to counter an impulse or urge to do something resulting in reactions that are hurtful to ones’ self or others. Currently, many conditions of behavioral addictions are very common, for example, compulsive buying, internet addiction, eating disorders, and gambling.^[3]^

Many smartphone users experience pain in the thumb/wrist, but whether those who develop pain are smartphone addicts or not has not been evaluated. Previous studies showed that using electronic devices or other devices that involve frequent use and movement of the thumb will lead to increasing load on the thumb, and therefore a higher prevalence of musculoskeletal disorders.^[4–6]^ Smartphone functions represent great potential for applications in medical education, as they allow doctors and students to access resources efficiently to support better decision making at the point-of-care.^[7–10]^ Despite their benefits, excessive use could result in various physical effects such as neck or wrist pain, and may be associated with disturbances of sleep and anxiety.^[11,12]^

In previous studies, there is a clear lack of research investigating this topic, and none of the previous investigations have been performed in Saudi Arabia. The aim of this study, therefore, was to evaluate the association between smartphone addiction and thumb/wrist pain and to determine the severity of the pain, as well as to calculate the prevalence of De Quervain tenosynovitis among medical students at King Abdulaziz University (KAU) in Jeddah.

## 2 Methods

A cross-sectional survey (level IV evidence) was conducted in May 2016. Participants were selected from undergraduate medical students at KAU, Jeddah, KSA. By using the standard formula to calculate sample size by prevalence, the calculated sample size was 385.^[13]^ Since no recent, accurate data were available, the prevalence was taken at 50%, with a 95% confidence interval and 5% marginal error. Study participants were recruited according to their academic year, and an equal percentage was obtained from each year. Men and women were divided equally among the academic year groups. All students between the ages of 18 and 25 years who owned a smartphone and use it for >2 hours daily were included. Individuals with a history of hand surgery or inflammatory arthritis were excluded.

The smartphone addiction scale short-version (SAS-SV) was translated by The Research unit in the English Language Institute at KAU. The English Language Institute is fully accredited by the United States based Commission for English Language Program Accreditation (CEA). Forward and backward translation was applied. In order to test the validity and reliability of the Arabic version, we administered the Arabic version to 100 students. We then administered the English version to the same students. Cronbach *α* and confirmatory factor analysis were applied to test the questionnaire.

SAS-SV was used to divide participants into the smartphone addict or non-addict groups according to their scores.^[14]^ SAS-SV was originally developed from an older version termed the smartphone addiction scale (SAS). It consists of 10 questions that are based on a self-reporting system with a Likert scale of 6 points (1: strongly disagree, 2: disagree, 3: weakly disagree, 4: weakly agree, 5: agree, 6: strongly agree). A cut-off value was reported as 31 for men and 33 for women.

Pain was assessed using the Arabic version of the patient-rated wrist and hand evaluation (PRWHE-A).^[15]^ The PRWHE-A was designed to assess pain and functional difficulties of the wrist and hand. It is a self-reported questionnaire that consists of 15 items. It is further divided into 2 subscales, the pain subscale (5 items) and function subscale (10 items). Each item is analyzed with a numerical scale (0–10) where (0) means no pain and (10) means the worst pain ever felt. The scoring system is calculated by adding the functional scores and dividing them by 2, then adding the pain scores to give a total of 100 points.^[16]^ Lower scores indicate better function and less pain.

For those who had experienced pain in the thumb or wrist of the dominant hand, the Finkelstein test was performed. The participants were asked to clench a fist with the thumb inside, and the examiner passively deviates it to the ulnar side; tenderness was checked at the radial styloid.^[17,18]^

Informed written consent was obtained from the participants. This study was approved by the Institutional Review Board of KAU (approval number 135–16) before conducting the survey. Data entry and analysis were conducted via SPSS (Statistical Package for the Social Sciences, IBM, New York, NY) version 24. Categorical variables are expressed as frequency or proportion. Continuous variables are expressed as median and interquartile range after testing the normality of the distribution using the Kolmogorov–Smirnov test for non-normally distributed data. The chi-square test was used to determine the association between categorical variables. The Mann–Whitney *U* test was used for comparison of non-parametric data between groups. A *P* value <.05 was considered significant.

## 3 Results

The total number of participants in this study was 387, with a response rate of 84%, including 204 (52.3%) men and 183 (47.7%) women (Table 1). Most of the participants were right-handed (92%). To verify the validity of the SAS-SV questionnaire, the correlation coefficients were calculated between each of the 10 questions, in which items 1 to 10 are the 10 questions in English language (Supplemental Digital Content [Appendix 1]) and 11 to 20 are the same 10 items but in Arabic language (Supplemental Digital Content [Appendix 2]). Results, reported in Table 2, show a significant correlation, where *P* < .001 for all items. A reliability analysis was carried out concerning the knowledge part of the questionnaire, comprising 10 items. Cronbach *α* showed the questionnaire to reach high reliability, *α* = 0.890. Most items appeared to be worthy of retention, resulting in a decrease in the alpha if deleted, as shown in Table 3.

**Table 1:**
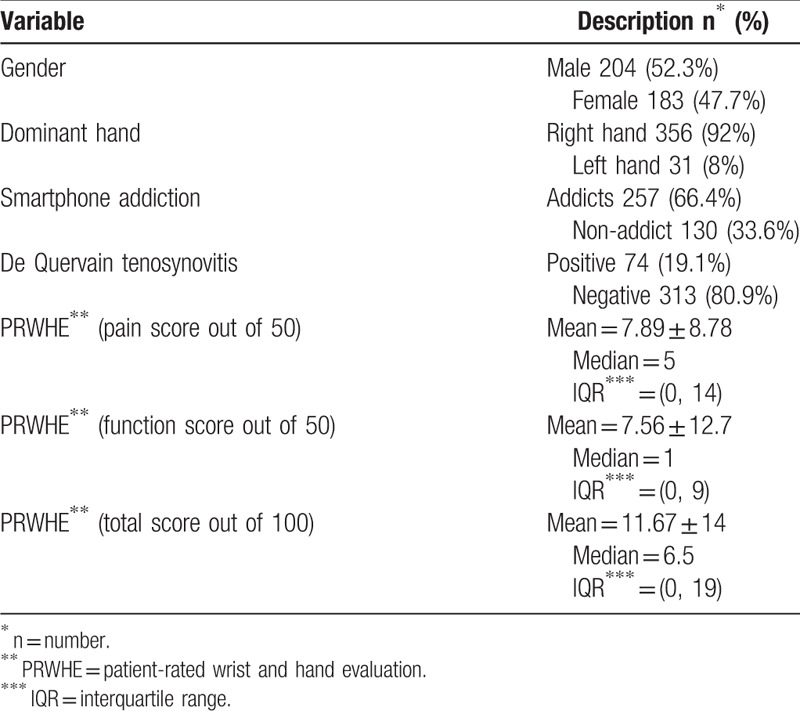
General characteristic of participants.

**Table 2:**
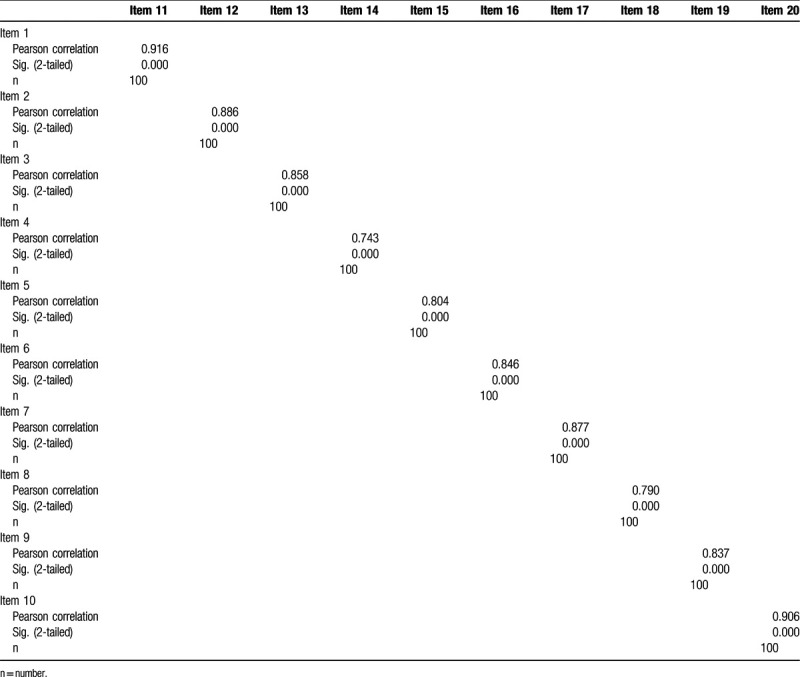
Correlation coefficients between each statement of the domains in Arabic and English showing significant correlation, *P* < .0001.

**Table 3:**
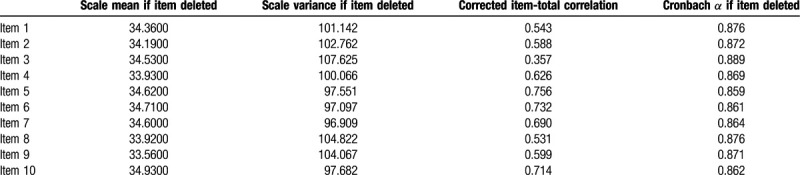
Cronbach *α* showing high reliability, *α* = 0.890.

According to the SAS-SV results, 257 (66.4%) participants were considered to be smartphone addicts, while 130 (33.6%) were deemed to be non-addicts. In total, 20.4% of participants reported pain in the thumb/wrist, but only 74 (19.1%) participants had a positive Finkelstein test, including 22 (10.8%) men and 52 (28.4%) women. The Mann–Whitney *U* test indicated that the PRWHE score (Table 4) was greater for smartphone addicts (median = 8, interquartile range = 0, 20) than for non-smartphone addicts (median = 4.25, interquartile range = 0, 13; *U* = 14,566.50, *P* = .036). The chi-square of independence was calculated comparing the frequency of a positive Finkelstein test in smartphone addicts and non-smartphone addicts; the *P* value was not significant (χ^2^ (1) = 3.028, *P* = .082).

**Table 4:**
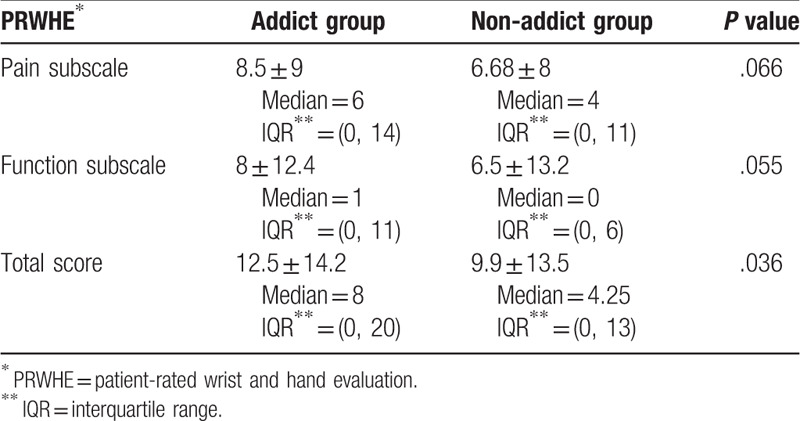
Mann–Whitney *U* test between PRWHE^∗^ scores and smartphone addiction scale-short version.

## 4 Discussion

The aim of this study was to evaluate the association between smartphone addiction and wrist/thumb pain and to determine the severity of the pain, as well as to calculate the prevalence of De Quervain tenosynovitis among medical students at KAU in Jeddah. There has been a rapid increase in smartphone use due to its multiple benefits, to the point that it has become a necessity. One study including 6 Asian countries showed that 62% of adolescents own smartphones.^[19]^ Another study in Switzerland reported that 97.6% of adolescents own smartphones.^[20]^

The results of the SAS-SV revealed that the majority of the participants were addicts (66.4%). The prevalence of smartphone addiction has been rising throughout the world. In Switzerland, the prevalence of smartphone addiction among adolescents was reported to be 16.9%.^[20]^ In a study from Riyadh, 76% of participants showed a moderate to high risk of smartphone and internet addiction.^[21]^ A German study enrolling resident doctors found that 27.1% were smartphone addicts. This study found that the use of smartphones was necessary to communicate with their families during the long hours of duty.^[22]^ An Indian meta-analysis found that the percentage of smartphone addicts ranged from 39% to 44%, and reported that smartphone usage is increasing because it is becoming a fundamental way to spend free time.^[23]^ People who are addicted to smartphones will probably have physical and psychosocial problems as well as internet addiction.^[24]^

Many adolescents report various musculoskeletal disorders due to the use of electronic devices.^[4,6,25]^ In the current study, 20.4% of all participants reported pain in the thumb/wrist. In a study from China, 43.4% of participants experienced thumb/wrist pain due to the use of different electronic devices.^[6]^ In a study from Pakistan, 42% of adolescents reported pain in the thumb/wrist due to smartphone use.^[4]^

Our study found a significant correlation between smartphone addiction and thumb/wrist pain (*P* < .05). The median total pain and disability score of the PRWHE-A for addicts was 8.00 with an interquartile range of 0 to 20, meaning that the total score for the majority of addicts (75%) was ≤20. The median total pain and disability score of the PRWHE-A for non-addicts was 4.25 with an interquartile range of 0 to 13. Since the severity of the pain for those with thumb/wrist pain while using a smartphone and electronic devices has not been previously established, we compared our data with similar results reported using the PRWHE for different hand and wrist disorders. In Switzerland, the mean score of pain and disability was 11 for individuals with a distal radial fracture (DRF) after 6 months’ follow-up.^[26]^ In Canada, the mean score of pain and disability was 13.5 for those with DRF after 1 year follow-up.^[27]^ Usually, patients with DRF experience some mild pain and stiffness in this follow-up period.^[26,27]^

The thumb is usually involved in smartphone usage. There are many studies that have reported on De Quervain tenosynovitis and its association with different electronic devices.^[28–30]^ De Quervain tenosynovitis is characterized by pain in the wrist over the radiostyloid process.^[31]^ Overuse of the thumb for mobile texting have been considered a risk factor for De Quervain tenosynovitis.^[4,32–34]^ The most common finding is a positive Finkelstein test.^[35]^

Our study found that 19.1% of our participants had a positive Finkelstein test, including 10.8% of male participants and 28.4% of female participants. According to one study in Pakistan, the Finkelstein test was positive for almost one-half of the total study population who reported texting very often on their phones and smartphones (n = 149 out of 300).^[4]^ Another study in India showed similar results: the Finkelstein test was positive in 40% of participants.^[32]^ A French study conducted in 2006 reported that the prevalence of De Quervain tenosynovitis was 1.2%, with an incidence of 0.6% and 2.1% among men and women, respectively.^[36]^

Although we found that thumb/wrist pain was associated with smartphone addiction, there was no significant association between smartphone addiction and Finkelstein test results (*P* > .05). There are other potential causes of thumb/wrist pain, including the extensor pollicis longus in the third dorsal compartment, flexor pollicis longus, thenar eminence, and median nerve; additionally, other clinical and subclinical changes could be involved due to mobile phone use, as discussed and demonstrated by ultrasound in recent studies.^[12,32]^

## 5 Conclusion

Our study concluded that students who are heavy users of smartphone have mild pain and stiffness in thumb\wrist. Although positive Finkelstein test wasn’t associated with smartphone addiction, other clinical and subclinical changes in thumb\wrist soft tissues could cause pain. That was evident in the association that was found between higher PRWHE scores and heavy smartphone usage. Compared with previous papers estimating the prevalence of smartphones addiction, we had a higher prevalence which can indicate that the smartphone addiction is rising.

The effect of smartphone addiction must be further evaluated using more accurate diagnostic measurements to understand its effect on human beings in order to prevent it. Although we are proud with our sample size, our study had multiple other weaknesses. Data about the duration of using the smartphone, position while usage, general physical habits of the participants, and hobbies have not been collected. Information about other devices as well such as personal computers and tablets and how often are they used have not been taken into account in our study.

### 5.1 Limitations

Although we are satisfied with our sample size, our study had multiple other weaknesses. Data concerning the duration of smartphone use, position during usage, general everyday habits, hobbies, and how the phone is usually carried and used were not collected. Information about other devices, such as personal computers and tablets, and how often are they were used was also not collected. Additionally, attendance at a gym and heavy training including weight lifting might affect the prevalence of this condition.

## Acknowledgments

The authors thank Min Kwon, Fatmah N. Hasani, and Hussam Rajab for assisting and providing the questionnaires used in this research and Abdulmalik Abumohssin for comments and revisions that greatly improved the manuscript. They would like to thank Editage (www.editage.com) for English editing, manuscript style and structure, and technical review.

## Author contributions

**Conceptualization:** Ayman Baabdullah, Diyaa Bokhary, Yousof Kabli, Omar Saggaf, Motaz Daiwali, Amre Hamdi.

**Data curation:** Ayman Baabdullah, Diyaa Bokhary, Yousof Kabli, Omar Saggaf, Motaz Daiwali, Amre Hamdi.

**Formal analysis:** Ayman Baabdullah, Amre Hamdi.

**Investigation:** Ayman Baabdullah, Diyaa Bokhary, Yousof Kabli, Omar Saggaf, Motaz Daiwali, Amre Hamdi.

**Methodology:** Ayman Baabdullah, Diyaa Bokhary, Yousof Kabli, Omar Saggaf, Motaz Daiwali, Amre Hamdi.

**Project administration:** Ayman Baabdullah, Diyaa Bokhary, Yousof Kabli, Motaz Daiwali, Amre Hamdi.

**Resources:** Amre Hamdi.

**Supervision:** Ayman Baabdullah, Diyaa Bokhary, Amre Hamdi.

**Validation:** Ayman Baabdullah, Omar Saggaf, Amre Hamdi.

**Visualization:** Amre Hamdi.

**Writing – original draft:** Ayman Baabdullah, Diyaa Bokhary, Yousof Kabli, Omar Saggaf, Motaz Daiwali, Amre Hamdi.

**Writing – review & editing:** Ayman Baabdullah, Diyaa Bokhary, Yousof Kabli, Omar Saggaf, Motaz Daiwali, Amre Hamdi.

Ayman Baabdullah orcid: 0000-0002-5784-0241.

## Supplementary Material

Supplemental Digital Content

## Supplementary Material

Supplemental Digital Content
