# Supplementary material for: The association between smartphone addiction and thumb/wrist pain: A cross-sectional study
Source: Medicine (Baltimore). 2020 Mar 6;99(10):e19124. doi: 10.1097/MD.0000000000019124 (PMC7478614; doi:10.1097/MD.0000000000019124)
Supplement: Supplemental Digital Content [file medi-99-e19124-s001.docx]

**Supplemental Digital Content (Appendix 1). English Version of SAS-SV**

| **Items** | **Statements** | **Strongly disagree** | **Disagree** | **Weakly disagree** | **Weakly agree** | **Agree** | **Strongly agree** |
| --- | --- | --- | --- | --- | --- | --- | --- |
| 1 | Missing planned work due to smartphone use | 1 | 2 | 3 | 4 | 5 | 6 |
| 2 | Having a hard time concentrating in class, while doing assignments, or while working due to smartphone use | 1 | 2 | 3 | 4 | 5 | 6 |
| 3 | Feeling pain in the wrists or at the back of the neck while using a smartphone | 1 | 2 | 3 | 4 | 5 | 6 |
| 4 | Won’t be able to stand not having a smartphone | 1 | 2 | 3 | 4 | 5 | 6 |
| 5 | Feeling impatient and fretful when I am not holding my smartphone | 1 | 2 | 3 | 4 | 5 | 6 |
| 6 | Having my smartphone in my mind even when I am not using it | 1 | 2 | 3 | 4 | 5 | 6 |
| 7 | I will never give up using my smartphone even when my daily life is already greatly  affected by it. | 1 | 2 | 3 | 4 | 5 | 6 |
| 8 | Constantly checking my smartphone so as not to miss conversations between other  people on Twitter or Facebook | 1 | 2 | 3 | 4 | 5 | 6 |
| 9 | Using my smartphone longer than I had intended | 1 | 2 | 3 | 4 | 5 | 6 |
| 10 | The people around me tell me that I use my smartphone too much | 1 | 2 | 3 | 4 | 5 | 6 |
