# Supplementary material for: The association between smartphone addiction and thumb/wrist pain: A cross-sectional study
Source: Medicine (Baltimore). 2020 Mar 6;99(10):e19124. doi: 10.1097/MD.0000000000019124 (PMC7478614; doi:10.1097/MD.0000000000019124)
Supplement: Supplemental Digital Content [file medi-99-e19124-s002.docx]

**Supplemental Digital Content (Appendix 2). Arabic Version of SAS-SV**

**البيانات التالية تتعلق بإدمان استخدام الهواتف الذكية**

| **أوافق بشدة** | **أوافق** | **أوافق بدرجة بسيطة** | **لا أوافق بدرجة بسيطة** | **لا أوافق** | **لا أوافق بشدة** | **البيان** | **الرقم** |
| --- | --- | --- | --- | --- | --- | --- | --- |
| **6** | **5** | **4** | **3** | **2** | **1** | **عدم إكمال الأعمال المخطط لها بسبب استخدام الهاتف الذكي** | **1** |
| **6** | **5** | **4** | **3** | **2** | **1** | **وجود صعوبة في التركيز في الصف، أو حين إكمال المهمة، أو أثناء العمل بسبب استخدام الهاتف الذكي.** | **2** |
| **6** | **5** | **4** | **3** | **2** | **1** | **الشعور بألم في الرسغين أو في الجزء الخلفي من الرقبة أثناء استخدام الهاتف الذكي** | **3** |
| **6** | **5** | **4** | **3** | **2** | **1** | **لا أتحمل عدم وجود هاتف ذكي لدي** | **4** |
| **6** | **5** | **4** | **3** | **2** | **1** | **الشعور بالتوتر العصبي وعدم الصبر عندما لا يكون الهاتف الذكي في يدي** | **5** |
| **6** | **5** | **4** | **3** | **2** | **1** | **وجود هاجس الارتباط مع هاتفي الذكي حتى عندما لا أقوم باستخدامه** | **6** |
| **6** | **5** | **4** | **3** | **2** | **1** | **أنا لن أتخلى أبداً عن استخدام هاتفي الذكي مع ما له من تأثيرات سلبية على مسار حياتي اليومية.** | **7** |
| **6** | **5** | **4** | **3** | **2** | **1** | **أنا أستخدم هاتفي الذكي وأتحقق منه باستمرار وذلك حتى لا تضيع علي المحادثات مع الأشخاص الآخرين على تطبيقات تويتر، او فيسبوك** | **8** |
| **6** | **5** | **4** | **3** | **2** | **1** | **أستخدم هاتفي الذكي لا شعوريا أكثر مما أنوي.** | **9** |
| **6** | **5** | **4** | **3** | **2** | **1** | **معارفي ممن حولي يقولون لي أنني أستخدم الهاتف الذكي أكثر من اللازم** | **10** |
